# The impact of strategic napping on peak expiratory flow and respiratory function in young elite athletes

**DOI:** 10.1186/s13102-024-00842-4

**Published:** 2024-02-09

**Authors:** Ahmet Kurtoğlu, Özgür Eken, Engin Aydın, Bekir Çar, Hadi Nobari

**Affiliations:** 1 Department of Coaching Education, Faculty of Sport Science, Bandirma Onyedi Eylul University, Balıkesir, 10200 Turkey; 2 Department of Physical Education and Sport Teaching, Faculty of Sports Sciences, Inonu University, Malatya, 44280 Turkey; 3 Department of Pediatrics, Zeynep Kamil Maternity and Children’s Disease Training and Research Hospital, University of Health Sciences, Istanbul, 34668 Turkey; 4 Department of Physical Education and Sport Teaching, Faculty of Sport Sciences, Bandirma Onyedi Eylul University, Balıkesir, 10200 Turkey; 5 Faculty of Sport Sciences, University of Extremadura, Cáceres, 10003 Spain; 6 Department of Exercise Physiology, Faculty of Educational Sciences and Psychology, University of Mohaghegh Ardabili, Ardabil, 56199-11367 Iran

**Keywords:** Athletic performance, Strategic napping, Respiratory function, Peak expiratory flow, Sleep, Young elite athletes

## Abstract

Respiratory health is a critical determinant of athletic performance, and the utilization of restorative strategies, such as strategic napping, may offer a competitive edge to athletes. This study investigates the effects of nap duration on the respiratory function of young elite athletes who have achieved top rankings national competitions. Participants engage in three test sessions with varying nap durations: no nap (N0), a 25-minute nap (N25), and a 45-minute nap (N45), with a minimum 72-hour interval between sessions. Respiratory parameters including Forced Vital Capacity (FVC), Forced Expiratory Volume in one second (FEV1), FEV1/FVC ratio, Peak Expiratory Flow rate (PEF), Forced Expiratory Flow at 25–75% of FVC (FEF25-75%), and Forced Expiratory Time (FET) are assessed. Results reveal a significant enhancement in PEF values following a 45-minute nap (N45) compared to the no-nap control (N0) [F_1 − 11_=7.356, *p* =.004, η_p_^2^ = 0.401, (95% CI for difference: -1.56 to − 0.056)], indicating a potential positive influence of napping on maximum expiratory flow rate and, consequently, athletes’ respiratory performance. While no significant changes are observed in other respiratory parameters across different nap durations, these findings underscore the potential benefits of strategic napping in optimizing respiratory health in young elite athletes.

**Supplementary Information:**

The online version contains supplementary material available at 10.1186/s13102-024-00842-4.

## Background

In sports science, elite young athletes are continuously seeking methods to gain a competitive advantage, with recent focus on the role of sleep in optimizing key physiological functions essential for athletic excellence. Sleep is a crucial factor that significantly influences performance outcomes [1]. Sleep is a fundamental physiological process with wide-ranging benefits, crucial for physical development, emotional regulation, and cognitive functions [2]. Moreover, sleep plays a key role in regulating molecular mechanisms and maintaining metabolic balance [3]. Waterhouse et al. [4] suggest that a restorative night’s sleep can rejuvenate, boost cognitive abilities, and alleviate fatigue. This viewpoint is complemented by the findings of Leeder et al. [5], highlight the importance of sufficient sleep for optimal performance and recovery. Acknowledging sleep’s vital role emphasizes the need to maintain and promote healthy sleep patterns, especially where optimal performance and well-being are crucial.

In response to this demand for enhanced sleep, daytime napping has emerged as a strategic tool employed by athletes to augment both the quality and quantity of their sleep [6]. Notably, it has been observed that adequate sleep is pivotal for supporting nocturnal sleep patterns and optimizing physical performance. This is particularly pertinent because the conventional recommendation of 8 h of sleep per day may fall short of fulfilling the unique sleep requirements of athletes, given the substantial demands they face, both physically and mentally [1, 7–11].

Napping represents a widespread and significant behavioral phenomenon with notable implications for public health, serving as a vital strategy to mitigate the adverse consequences associated with insufficient sleep, both in the short and long term [12]. Defining a nap in precise terms, it constitutes any episode of sleep characterized by a duration falling below 50% of an individual’s typical, major sleep period [13]. These naps, commonly referred to as ‘short sleeps,’ are markedly shorter than an individual’s customary nightly sleep episode [13, 14].

A noteworthy consideration often overlooked in the sleep literature pertains to the practice of athletes strategically integrating naps into their routines, driven by the belief that napping may yield benefits in terms of recovery and performance enhancement [15, 16]. Daytime napping, if effectively employed, can augment the total quantity of sleep acquired within a 24-hour timeframe. Many athletes have reported incorporating daytime naps as a deliberate component of their training programs. For instance, a study conducted by Sargent et al. [15] revealed a greater frequency of napping on training days compared to rest days. Consequently, an athlete’s daily regimen should encompass both well-timed daytime napping and nocturnal sleep hygiene practices to optimize their sleep and performance outcomes. While a substantial body of research has delved into the effects of manipulating nighttime sleep duration on various aspects of athletic performance, such as sprint times and reaction time [17], there has been relatively less emphasis on investigating the impact of napping [18, 19].

Moreover, scientific investigations have illuminated the positive impact of napping opportunities on various facets of athletic performance. These beneficial effects encompass enhancements in short-term maximal performance, attention, alleviation of feelings of muscle soreness and fatigue, reduction in stress levels, improvements in vigilance, shuttle run performance, the 5-meter Shuttle Run Test, repeated sprint performance, and sprint performance [9, 10, 20–22]. The duration of nap opportunities, ranging from 20 to 90 min, has been explored, further underscoring the versatility of napping as an effective strategy to bolster athletic prowess.

In addition to this information, some epidemiologic studies have reported conflicting results between daytime sleepiness and mortality risk [23]. Leng et al. reported that excessive napping negatively affected respiratory function in sedentary individuals under 65 years of age according to the findings obtained from 13 years of observation [24]. Another study by Masa et al. argued that napping may be an indicator of sleep apnea and may be responsible for the cardiovascular diseases observed in these individuals [25].

Botonis et al. (2021) highlight mixed results in studies on napping’s impact on athletic performance, emphasizing that benefits from mid-day naps vary depending on the nap duration and factors like sleep inertia and time between napping and performance testing [26]. Similarly, Lastella et al. (2021) indicate that napping, especially between 20 and 90 min during early to mid-afternoon, can enhance athletes’ physical and cognitive performance, reduce fatigue, and improve psychological state and night-time sleep [27]. Furthermore, Souabni et al. (2021) suggest that diurnal napping, particularly around 90 min, can counteract the negative effects of partial sleep deprivation in athletes, enhancing both physical and cognitive performance, although these findings need careful consideration due to study quality and biases [28]. Our study delves into the intricate relationship between nap timing and its impact on the respiratory functions of elite young athletes. Extending beyond the established research on napping’s effects on performance metrics, we aim to explore the uncharted aspects of this restorative strategy’s potential benefits in respiratory health. By doing so, this research contributes to the evolving discourse within sports science, highlighting the importance of considering comprehensive physiological parameters, including respiratory functions, when assessing the efficacy of strategic napping in athletic contexts. The selection of 25-minute and 45-minute nap durations in the study on elite athletes’ respiratory function is grounded in sleep science and practicality. These durations align with sleep cycle phases, avoiding deep sleep to prevent sleep inertia—a state of cognitive impairment post-awakening. The 25-minute duration is chosen to avoid deep sleep and minimize sleep inertia, as supported by studies suggesting short naps are beneficial for alertness and mood without inducing grogginess [29]. The 45-minute duration, in contrast, is potentially long enough to complete a full sleep cycle, offering more comprehensive restorative benefits, including enhanced cognitive function [30]. These durations also align with the tight schedules of elite athletes, providing a feasible and effective strategy for incorporating naps into their routines [15, 19, 27, 31]. Our study extends the application of these durations from general cognitive and physical performance, explored in prior research [27, 32–34], to the specific context of respiratory functions in athletes, a novel and under-researched area in sports science [35, 36].

Napping may enhance respiratory function in athletes through several mechanisms, such as improved autonomic regulation during sleep, which can lead to a reduction in stress and inflammation, potentially benefiting respiratory health [37, 38]. Additionally, short periods of sleep, like naps, may facilitate better oxygenation and removal of metabolic by-products, thus enhancing lung function [39]. While the beneficial effects of napping on cognitive and physical performance are well-established [14, 28, 32], our study aims to bridge the gap in research by focusing on the impact of napping on respiratory functions, an area that has not been extensively explored in elite athletes [40]. This approach not only reinforces the multifaceted benefits of napping but also contributes valuable insights to the domain of sports science, particularly in optimizing athlete health and performance through holistic strategies [41, 42].

This study posits two hypotheses to investigate the impact of nap duration on the respiratory function of young elite athletes who have excelled in national competitions. Firstly, we hypothesize that there will be a significant improvement in Peak Expiratory Flow (PEF) values following a 45-minute nap (N45) compared to the no-nap control (N0). Secondly, we anticipate that other respiratory parameters, including Forced Vital Capacity (FVC), Forced Expiratory Volume in one second (FEV1), FEV1/FVC ratio, Forced Expiratory Flow at 25–75% of FVC (FEF25-75%), and Forced Expiratory Time (FET), will exhibit significant changes across different nap durations, indicating potential benefits of strategic napping in optimizing respiratory function among young elite athletes. These hypotheses aim to shed light on the potential advantages of strategic napping as a tool for enhancing respiratory health and athletic performance in this specific athlete population.

## Method

### Participants

This approach was fundamental in upholding the research’s integrity and the reliability of the collected data. The minimum sample size for the present study was calculated using G-power software 3.1.9.7. (University of Dusseldorf, Dusseldorf, Germany) [43]. According to this analysis; F tests (ANOVA: repeated measures, within factors were used to calculate power following the design of our study; within factors; α err prob = 0.05; minimum effect size = 0.45 and power (1-β err prob) = 0.80 (actual power = 81.2%). The power analysis indicated that the study should have at least 10 participants in total. Due to the risk of participants leaving, 16 young elite athletes were chosen in all. Because they did not complete all of the required sessions, four of the young elite athletes (*n* = 4, 25%) were disqualified from the data analysis.

In this study, 12 young elite athletes participated. Their ages ranged from 12.50 ± 1.31 years, their height ranged from 164.91 ± 8.67 cm, their body mass ranged from 44.83 ± 8.49 kg and their body mass index ranged from 16.34 ± 1.67 kg/m^2^. These participants were selected from two specific disciplines, high jump (25%, 4 individuals), and short-distance running (75%, 8 individuals), based on their achievements of ranking within the top 5 positions in their respective age categories in national championships, following the criteria established by Matsudo et al. in 1987 [44]. To ensure the relevance and suitability of participants for our research, the following inclusion criteria were applied: (a) participants who had achieved a top 5 ranking in national competitions organized by the Turkish Athletics Federation, (b) being registered in the database of the Turkish Athletics Federation, and (c) Participants engaging in training sessions for a minimum of three days per week [45]. Exclusion criteria were applied to ensure the research’s internal validity and the robustness of its outcomes. Consequently, participants who met any of the following criteria were excluded from the study: (a) presence of respiratory function disorders, (b) cardiac problems, (c) tachycardia and bradycardia, (d) hematological issues, (e) active infections, (f) chronic obstructive pulmonary disease, (g) asthma, (h) use of exogenous substances affecting respiratory functions, (i) other health issues impacting respiratory functions, (j) hyperactivity. Furthermore, participants who did not heed researcher instructions, encountered challenges in adhering to study protocols, or experienced difficulties in complying with guidance during the research were also excluded from the study. The strict application of these inclusion and exclusion criteria was instrumental in ensuring that the study’s participant pool consisted exclusively of young elite athletes without significant respiratory or other health issues that could potentially confound research outcomes. Besides the participants were not habitual nappers.

This research was conducted in accordance with the principles outlined in the Helsinki Declaration. The study’s objectives, rationale, and hypotheses were explained to the participants, and their informed consent was obtained as a result. Since the participants were under the age of 18, parental consent was also obtained. Furthermore, to ensure the adherence of this research to ethical principles, the necessary approvals were obtained from the Bandirma Onyedi Eylul University, Non-Interventional Health Sciences Research Ethics Committee, under decision number 2023-25. This oversight ensured that the study was conducted in accordance with established ethical standards and guidelines.

### Experimental design

**Fig. 1:**
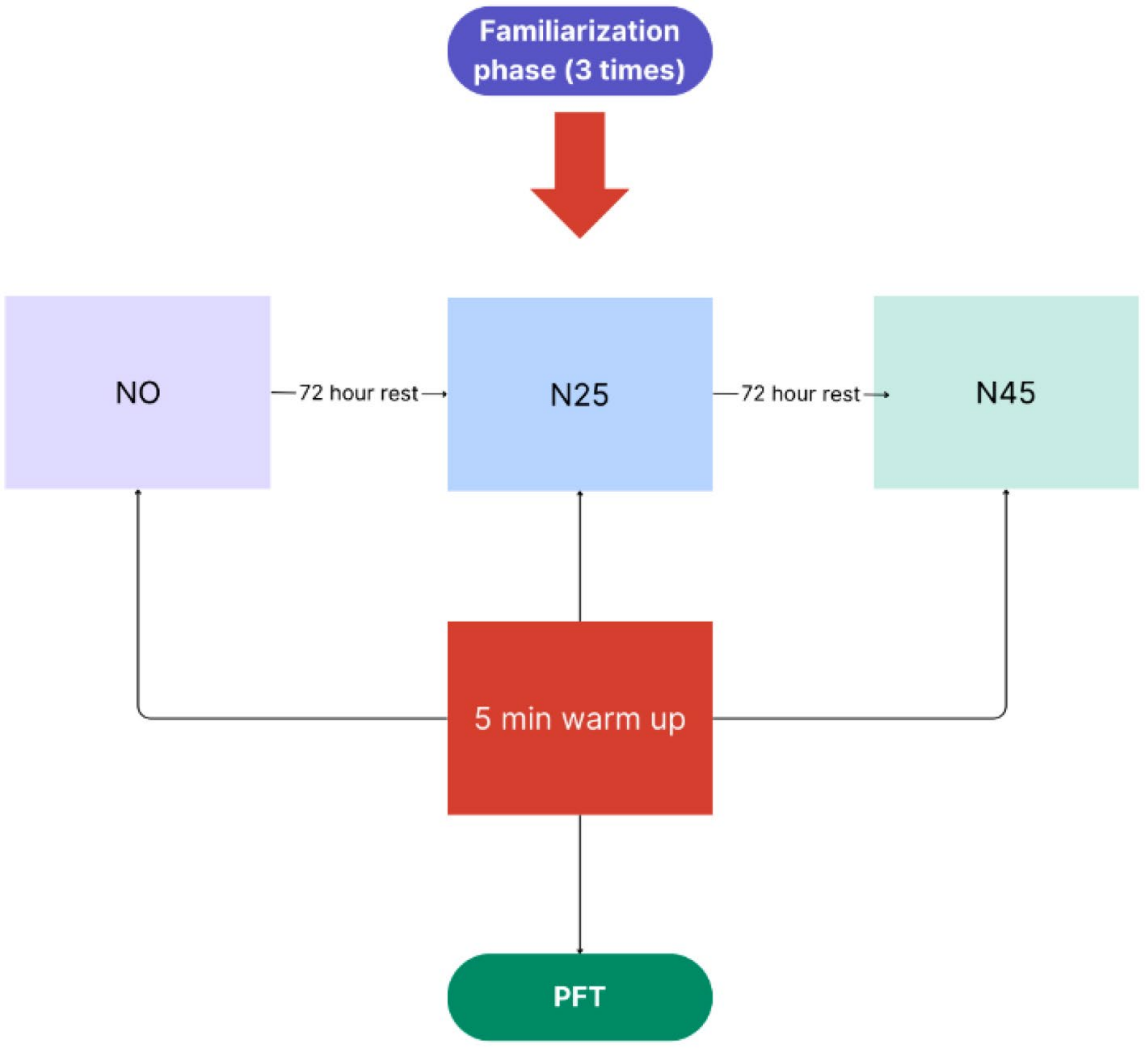
Experimental design of study

Prior to commencing the study, three familiarization sessions were conducted to ensure the athletes’ familiarity with both the designated nap location and the Pulmonary Function Test (PFT) procedures following napping. Subsequently, participants arrived at the laboratory for the three specified test sessions [No nap opportunity (N0), 25 min nap opportunity (N25), and 45 min nap opportunity (N45)] with a minimum of 72 h between sessions. Upon arrival at the laboratory, participants were allotted ten minutes for acclimatization to their sleeping environment. At 1:40 p.m., participants were instructed to select their preferred lying position. Commencing at 2:00 p.m., participants were granted an opportunity for N0, N25, and N45 protocols within dark and quiet sleeping chambers. During the nap period, participants in all conditions (N0, N25, and N45) refrained from engaging in routine and visual activities, such as using cell phones and playing video games. This is because numerous studies in the literature have demonstrated that visual activities can impact nap quality [46, 47]. After the strategic nap, participants completed a standard warm-up routine consisting of two minutes of light jogging followed by three minutes of specific exercises including foot crawls, toe and ankle rotations, trunk side stretches, trunk rotator stretches, hip circles and knee bends. Following the warm-up, participants underwent a Pulmonary Function Test (PFT) (Fig. 1).

### Data collection

#### Anthropometric measurements

Volunteers SECA® (Gmbh, Hamburg, Germany) were assessed in a standing position while barefoot, with their ankles, calves, hips, scapula, and head positioned against a wall. To ensure accuracy, the Frankfurt model was employed to determine head position, with height measurements taken during inhalation. Participants were instructed to wear lightweight clothing (Toledo 2096 PP, São Bernardo do Campo, Brazil) when body mass was assessed. The Body Mass Index (BMI) was calculated by dividing the participant’s weight (in kilograms) by the square of their height in meters [48]. For the reliability of the participants’ respiratory function, heart rate (HR) was measured with a validated and reliable Fitbit Charge 3 smart wristband for the detection of tachycardia and bradycardia [49, 50].

#### Pulmonary functional test (PFT)

In this study, respiratory functions were assessed using the MIR Minispir spirometer device (Italy). Prior to testing, participants’ nasal passages were cleaned, and they were comfortably seated on a chair. The Pulmonary Function Test (PFT) was then conducted. During the Respiratory Function Testing (RFT), participants’ nostrils were blocked, and they were instructed to securely hold a disposable mouthpiece to prevent any air leakage. Participants were closely guided by the responsible researcher and followed the respiratory control prompts displayed on the device’s screen. They were directed to take small breaths until signaled by the device. Once the device indicated the end of inhalation time, participants performed a forced expiration until another signal was received from the device. Each participant underwent two repetitions of the test, and the best value obtained was recorded for analysis. The following parameters were recorded as part of the RFT: Forced Vital Capacity (FVC), Forced Expiratory Volume in one second (FEV1), FEV1/FVC ratio, Forced Expiratory Flow at 25–75% of FVC (FEF25-75%), and Peak Expiratory Flow rate (PEF). If the difference between FVC and FEV1 was less than 5%, a third test was not deemed necessary, following the guidelines established by Ranu et al. in 2011 and Xavier et al. in 2020 [51, 52]. This comprehensive assessment allowed us to accurately evaluate participants’ respiratory functions under standardized conditions.

#### Statistical analysis

In this study, SPSS software (IBM, version 25, Chicago) was employed for statistical analyses. A normality analysis of the data was conducted using the Shapiro-Wilk test, which confirmed that the data followed a normal distribution. To assess the homogeneity of variances, the Levene Test was performed. To analyze the respiratory functions at different nap protocols, a Repeated Measures ANOVA test was conducted. Additionally, to determine differences between tests, the Bonferroni post-hoc test was applied. The results of the ANOVA test were determined based on the Mauchly’s Test of Sphericity. If Mauchly’s Test of Sphericity yielded a value greater than 0.05, assumptions of sphericity were considered met; otherwise, the Greenhouse-Geisser correction was applied. Effect sizes were calculated using Cohen’s d formula to determine the magnitude of the findings. Effect size for ANOVA was determined based on partial eta squared (ηp2) values, with ηp2 values indicating the effect size as follows: ηp2 ≤ 0.01 indicating a small effect size, 0.01 ≤ ηp2 ≤ 0.06 indicating a medium effect size, and ηp2 ≥ 0.14 indicating a large effect size [53]. The significance level for this research was set at 0.05. Since the data were normally distributed, evaluations were presented as mean (M) and standard deviation (S.D.).

## Results

The demographic information of the participants. According to the table, the participants’ age was determined to be 12.50 ± 1.31 years, height 164.91 ± 8.67 cm, weight 44.83 ± 8.49 kg, and BMI value 16.34 ± 1.67 kg/m².

**Fig. 2:**
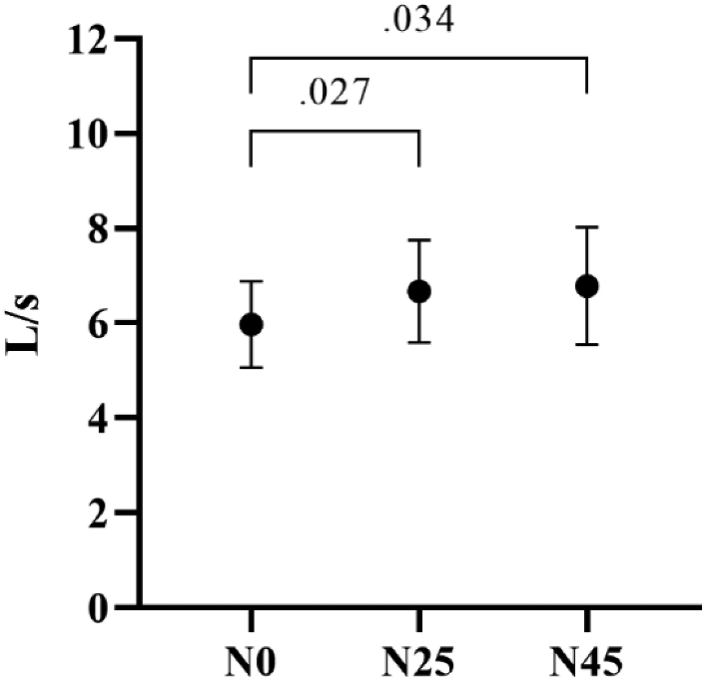
Comparison of participants’ N0, N25, and N45 PEF values

**Fig. 3:**
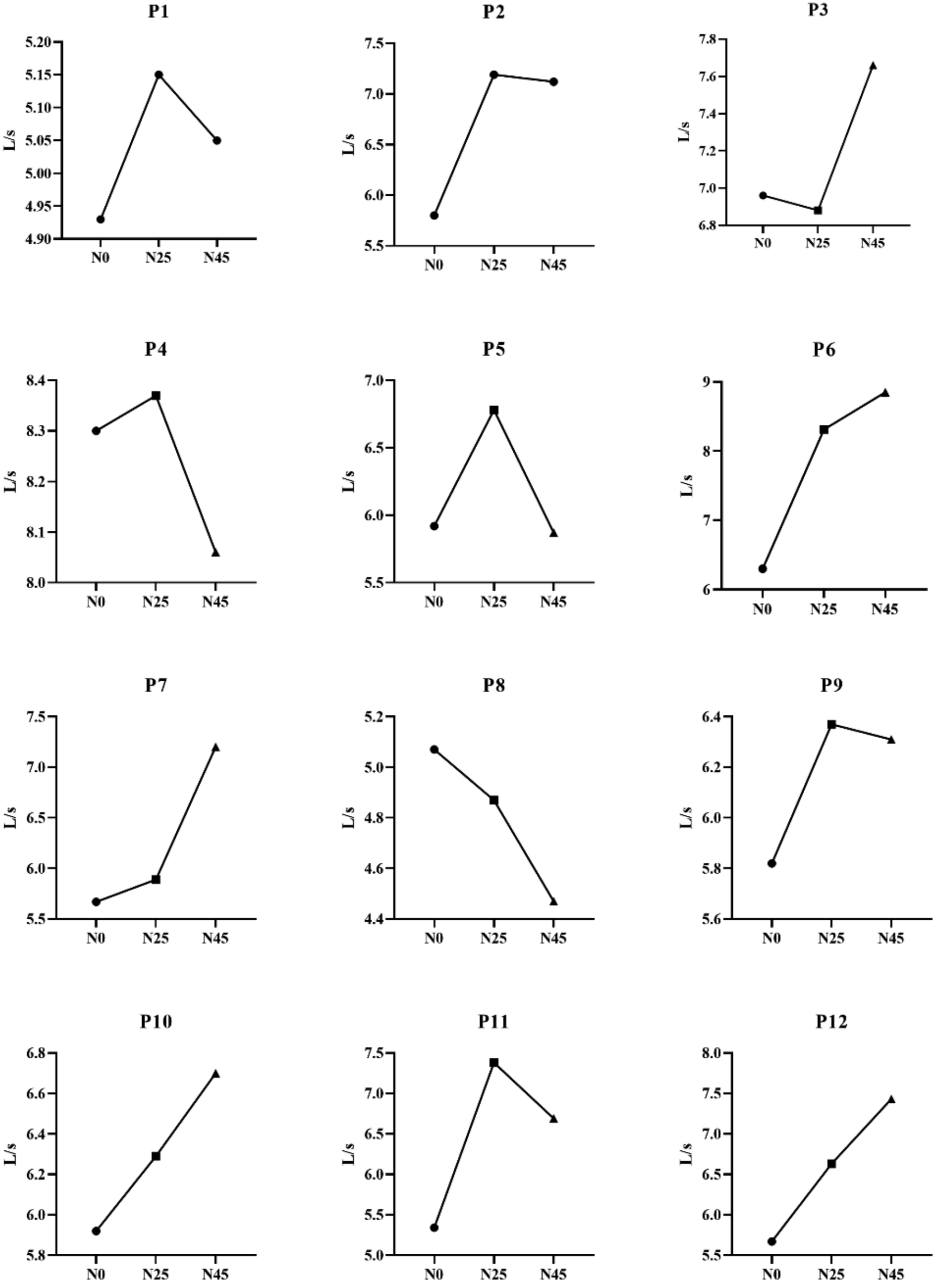
Individual PEF changes to different strategic nap procedures

Table 1 compares the resting respiratory functions of the participants in N0, N25, and N45. According to the table, the participants’ Peak Expiratory Flow (PEF) values significantly differed from N0 to N45 (F1-11 = 7.356, *p* =.004, ηp2 = 0.401). The post-hoc Bonferroni test revealed significant differences between the N0 and N45 PEF values (respectively; *p* =.034, [-1.563 to -0.56 95% CI], *p* =.027, [-1.326 to -0.76 95% CI]), while no significant difference was observed between N0 and N25 PEF values (*p* >.05) (Fig. 2). However, the participants’ Forced Vital Capacity (FVC), Forced Expiratory Volume in one second (FEV1), FEV1/FVC ratio, Forced Expiratory Flow at 25–75% of FVC (FEF25-75%), and Forced Expiratory Time (FET) values did not exhibit significant variations across different napping opportunity (*p* >.05). The changes in the individual PEF values of the participants are given in Fig. 3.

**Table 1:** Impact of nap duration on pulmonary function parameters

Parameters	Time	M ± S.D.	F	p	η_p_^2^	95% CI	
FVC (L)	N0	3.52 ± 0.71	2.428	0.136	0.181		
	N25	3.66 ± 0.80					
	N45	3.64 ± 0.78					
FEV1 (L)	N0	3.18 ± 0.62	2.769	0.110	0.356		
	N25	3.31 ± 0.69					
	N45	3.31 ± 0.69					
FEV1/FVC (%)	N0	90.60 ± 5.17	0.138	0.872	0.012		
	N25	91.06 ± 6.53					
	N45	91.05 ± 6.36					
PEF (L/s)	N0	5.97 ± 0.90	7.356	0.004*	0.401		
	N25	6.67 ± 1.07					
	N45	6.78 ± 1.23					
FEF25/75 (pred %)	N0	3.97 ± 1.00	0.195	0.707	0.147		
	N25	4.05 ± 1.02					
	N45	4.02 ± 1.12					
FET (sec)	N0	1.90 ± 0.51	0.018	0.982	0.002		
	N25	1.88 ± 0.66					
	N45	1.91 ± 0.56					

FVC: Forced Vital Capacity, PEF: Maximum Expiratory Flow Rate, FEV1: Forced Expiratory Volume İn 1 s, PEF: Peak Expiratory Flow, FET: Forced Expiratory Time, N0: No-nap control, N25: a 25-minute nap, N45: a 45-minute nap, * *p* <.05

## Discussion

Our study elucidates the intricate relationship between nap duration and respiratory functions in young elite athletes, underscoring the importance of strategic napping in enhancing athletic performance. The observed significant increase in Peak Expiratory Flow (PEF) values following a 45-minute nap, as opposed to the no-nap condition, highlights the potential of a 45-minute nap duration in boosting respiratory efficiency and performance. This enhancement in PEF posits a beneficial impact on maximal expiratory flow rate, a critical aspect of respiratory health in athletes [40, 54]. Conversely, our findings reveal that other respiratory parameters, including Forced Vital Capacity (FVC), Forced Expiratory Volume in one second (FEV1), the FEV1/FVC ratio, Forced Expiratory Flow at 25–75% of FVC (FEF25-75%), and Forced Expiratory Time (FET), did not exhibit significant alterations across different nap lengths. This outcome suggests that the beneficial effects of napping on respiratory functions might be more specific and nuanced, affecting certain aspects more than others [19, 40]. The lack of substantial change in PEF values between the no-nap condition and a 25-minute nap, alongside the absence of significant alterations in other respiratory metrics, indicates that nap duration is a pivotal factor in realizing respiratory benefits. Current literature extensively explores the enhancement in general performance metrics post-napping, but stops short of delving into the specific effects on respiratory mechanics, lung function, and respiratory muscle restoration [7, 26]. This gap highlights the novelty of our study within the domain of sports science, as it aims to bridge this lacuna by focusing on the relationship between a 45-minute nap duration and respiratory health in athletes. Our approach, therefore, not only aligns with the existing sleep literature but also extends it by exploring uncharted territories, particularly the implications of strategic napping on respiratory functions. This exploration is crucial for a comprehensive understanding of how napping, especially of specific durations like 45 min, can influence the multifaceted aspects of athletes’ health and performance, thereby emphasizing the importance and originality of our research in the broader context of sports science.

A substantial body of scholarly literature exists which encompasses research on the provision of napping opportunities [10, 11, 14, 20, 21, 55, 56]. However, no study has been found to examine its impact on respiratory functions. Hsouna et al. (2019) study investigated the effects of different nap durations on short-term maximal performance, attention, emotions, muscle soreness, fatigue, stress, and sleep. Twenty physically active men underwent various nap durations: no-nap (N0), 25 min (N25), 35 min (N35), and 45 min (N45). Results showed that N35 and N45 significantly improved 5-jump performance compared to N0. Attention was also better after N45. Participants reported lower fatigue, better sleep, and reduced stress after N25, N35, and N45 compared to N0. Emotions and fatigue remained unaffected [9]. In summary, while this study and our study explore the impact of napping on athletes, they address different aspects of athlete well-being and performance. The Hsouna et al. (2019) study investigates various napping durations and their effects on multiple parameters, while our study concentrates on the timing of naps and its impact on respiratory functions in elite young athletes. Boukhris et al. (2019) examined the impact of daytime naps of different durations on high-intensity, short-duration performance and perceived exertion. Seventeen physically active men participated in a 5-meter shuttle run test under four conditions: a 25-minute nap (N25), a 35-minute nap (N35), a 45-minute nap (N45), and a no-nap control (N0). The quality of each nap was rated on a scale from 0 (no sleep) to 10 (uninterrupted, deep sleep). Results showed that BD (best distance) increased significantly after N25 and N45 compared to N0, with N45 leading to the highest improvement. All three nap durations enhanced TD (total distance) compared to N0, with N45 again showing the most significant improvement. However, there were no significant differences in fatigue index (FI) among the nap durations and the no-nap condition. In terms of perceived exertion, RPE (rating of perceived exertion) was significantly lower after N45 compared to N25 and N0, indicating reduced perceived effort. Participants were able to fall asleep during all nap conditions, with reasonably good sleep quality scores. This study concludes that longer naps, especially the 45-minute nap, can enhance physical performance and reduce perceived effort during exercise [10]. But our study examined the effect different nap protocols on the respiratory functions of elite young athletes. It finds that certain respiratory parameters vary throughout the day, which can be useful for planning training programs.

Boukhris et al. (2023) examined the impact of a 40-minute nap opportunity (N40) on performance, muscle damage, inflammation, and subjective perceptions during a 5-meter shuttle run test (5msrt) involving fifteen male amateur athletes. They compared this condition to a no-nap scenario (NN). Blood biomarkers were analyzed to assess muscle damage (creatine kinase, lactate dehydrogenase, aspartate aminotransferase, alanine aminotransferase) and inflammation (C-reactive protein). Ratings of perceived exertion (RPE) were collected after each test repetition, while perceived recovery status (PRS) and delayed onset muscle soreness (DOMS) were evaluated five minutes post-exercise. The findings demonstrated that N40 significantly enhanced both the highest and total distances achieved during the 5msrt when compared to NN. Moreover, participants exhibited reduced levels of muscle damage and inflammation before and after exercise in the N40 condition. Notably, subjective perceptions of exertion (RPE), muscle soreness (DOMS), and recovery status (PRS) all favored N40 over NN [11]. Abdessalem et al. (2019) examined the effects of 25-minute daytime naps at different time slots on physical and mental performance in a study involving 18 physically active males. Four experimental conditions were employed: no-nap, naps at 13h00, 14h00, and 15h00, with all assessments conducted at 17h00. The study included a 5-meter shuttle run test measuring the highest distance (HD) and total distance (TD) covered, alongside the recording of the rating of perceived exertion (RPE) after each sprint. Additionally, vigilance was assessed using a digit cancellation test. The key findings highlighted a significant 4% improvement in TD at 17h00 following a nap at 14h00 in comparison to the no-nap condition and the nap at 13h00. Furthermore, HD demonstrated an 8% increase after the 14h00 nap compared to the no-nap condition, and a 7% increase following the 15h00 nap compared to the no-nap condition. Notably, HD was 6% higher after the 14h00 nap and 5% higher after the 15h00 nap compared to the 13h00 nap, which did not exert a discernible influence on physical performance at 17h00. Importantly, RPE and vigilance scores remained unaffected by the nap opportunities [21].

Waterhouse et al. (2007) investigated the effects of an afternoon nap on subjective alertness and performance in the context of partial sleep deprivation. The study involved ten healthy male participants with an average age of 23.3 years who underwent either a nap or a period of quiet rest from 13:00 to 13:30 h following a night of abbreviated sleep (sleep duration from 23:00 to 03:00 h only). Assessments were conducted thirty minutes after the afternoon intervention, which included measurements of alertness, short-term memory, intra-aural temperature, heart rate, choice reaction time, grip strength, and sprint times for both 2-meter and 20-meter sprints. The results of the study indicated several significant findings. The afternoon nap led to reductions in heart rate and intra-aural temperature. Notably, it improved alertness, reduced sleepiness, enhanced short-term memory, and increased accuracy in the 8-choice reaction time test. However, mean reaction times and grip strength remained unaffected. The most significant improvements were observed in sprint times, with the mean time for the 2-meter sprints decreasing from 1.060 s to 1.019 s, and the mean time for the 20-meter sprints decreasing from 3.971 s to 3.878 s [20]. In summary, the study’s findings suggest that an afternoon nap can effectively enhance alertness and various aspects of mental and physical performance in individuals experiencing partial sleep deprivation. The study by Souissi et al. (2020) investigated the effects of partial sleep deprivation (SDN) and a 30-minute nap opportunity on physical and cognitive performance, as well as mood states, in physically active students. They found that napping significantly improved vigilance and reaction time compared to not napping (no-Nap). This effect was observed in both normal sleep (NSN) and SDN conditions, with greater improvements during NSN. Participants displayed enhanced physical performance, with increased total and peak distances in the 5-meter shuttle run test, and a reduced fatigue index during the nap (Nap) compared to the no-nap condition (no-Nap). These improvements were consistent in both NSN and SDN situations, with NSN showing more significant enhancements. Napping was associated with reduced anxiety, fatigue, confusion, and depression scores, along with increased feelings of vigor. These mood improvements were evident in both NSN and SDN contexts, with NSN demonstrating more substantial enhancements [55]. These findings have implications for individuals in demanding physical activities, suggesting that strategic napping could help optimize performance and mood, particularly in scenarios where sleep is compromised.

Prior investigations have consistently noted that improvements in physical performance exhibit greater magnitude when naps exceeding a duration of 30 min are taken [14, 57]. Specifically, Hammouda et al. (2018) reported that, in the context of a running-based anaerobic sprint test, the highest power output, lowest power output, and mean power output were significantly elevated following a 90-minute nap compared to a 20-minute nap [57]. Furthermore, Lumley et al. (1986) documented that, subsequent to a night of complete sleep deprivation, naps spanning a duration of 60–120 min resulted in reduced levels of sleepiness among healthy participants in contrast to a 15-minute nap [58]. These findings underscore the salient role of nap duration in optimizing both physical performance and subjective alertness.

This study has several limitations that should be acknowledged. First, the relatively small sample size of 12 young elite athletes may limit the generalizability of the findings. Additionally, the fixed timing of afternoon naps may not align with individual circadian rhythms. Participant adherence to nap protocols and the absence of female athletes in the sample further affect the study’s external validity. A notable limitation of our study is the absence of objective sleep measurement tools during the napping interventions. We relied on participants’ subjective self-reports to assess sleep onset, duration, and quality, which could introduce biases and inaccuracies due to the subjective nature of self-assessment. Objective methods, such as polysomnography or actigraphy, would have provided more precise and detailed data on sleep architecture and quality, thereby enriching the validity of our findings. This limitation suggests that the results should be interpreted with caution, and future research should consider incorporating objective sleep measures to more accurately evaluate the impact of napping on athletic and physiological outcomes. Despite these limitations, this research provides valuable insights into the potential benefits of strategic napping on respiratory function in young elite athletes and suggests avenues for future investigations.

## Conclusion

In conclusion, our study offers valuable insights into the potential advantages of strategic napping for optimizing the respiratory health of young elite athletes. The notable improvement in PEF values suggests that strategic napping might positively affect the maximum expiratory flow rate and, consequently, respiratory performance. However, the underlying mechanisms of these effects, the ideal timing of naps, and their broader applicability to athletes across varying age groups and sporting disciplines necessitate further exploration. Despite the inherent limitations, our findings contribute to the evolving body of knowledge regarding sleep interventions in the context of athletic performance, emphasizing the potential role of napping as a valuable asset for athletes striving to enhance their respiratory health and overall competitive edge.

Practitioners, including coaches, sports scientists, and healthcare professionals, are encouraged to consider incorporating structured napping into athletes’ training regimens, especially during periods of high physical demand or in preparation for competitions. This study not only highlights the importance of sleep duration in athletic training but also underscores the need for a holistic approach to athlete health and performance enhancement. By integrating napping strategies tailored to individual athletes’ needs, practitioners can contribute to the improvement of respiratory parameters, thereby potentially enhancing overall athletic capacity and performance.

### Electronic supplementary material

Below is the link to the electronic supplementary material.

**Supplementary Material 1: Supplementary data 1**: We have included the supplementary data file labeled as Supplementary Data 1 in the manuscript. The legend for this database is appropriately provided for clarity within the manuscript.

**Supplementary Material 2: Supplementary table S1**: We have incorporated a supplementary table labeled as Table S1 in the.doc supplementary file, addressing the specific request. This table enhances the supplementary materials, and its label corresponds to the guidance provided for improved organization.

## Data Availability

Data are available for research purposes upon reasonable request to the corresponding author.
