# Supplementary material for: The impact of strategic napping on peak expiratory flow and respiratory function in young elite athletes
Source: BMC Sports Sci Med Rehabil. 2024 Feb 9;16:41. doi: 10.1186/s13102-024-00842-4 (PMC10854142; doi:10.1186/s13102-024-00842-4)
Supplement: Supplementary file 2 — Supplementary Material 2: Supplementary table S1: We have incorporated a supplementary table labeled as Table S1 in the.doc supplementary file, addressing the specific request. This table enhances the supplementary materials, and its label corresponds to the guidance provided for improved organization. [file 13102_2024_842_MOESM2_ESM.docx]

Table S1. provides key pulmonary function measurements (FVC, FEV1, PEF) among elite athletes, comparing no nap, 25-minute, and 45-minute nap effects.

| Parameters | Time | M±S.D. | F | p | η_p_^2^ | 95% CI | |
| --- | --- | --- | --- | --- | --- | --- | --- |
|  |  |  |  |  |  |  |  |
| FVC (L) | N0 | 3.52±0.71 | 2.428 | .136 | .181 |  |  |
|  | N25 | 3.66±0.80 |  |  |  |  |  |
|  | N45 | 3.64±0.78 |  |  |  |  |  |
| FEV1 (L) | N0 | 3.18±0.62 | 2.769 | .110 | .356 |  |  |
|  | N25 | 3.31±0.69 |  |  |  |  |  |
|  | N45 | 3.31±0.69 |  |  |  |  |  |
| FEV1/FVC (%) | N0 | 90.60±5.17 | .138 | .872 | .012 |  |  |
|  | N25 | 91.06±6.53 |  |  |  |  |  |
|  | N45 | 91.05±6.36 |  |  |  |  |  |
| PEF (L/s) | N0 | 5.97±0.90 | 7.356 | .004* | .401 |  |  |
|  | N25 | 6.67±1.07 |  |  |  |  |  |
|  | N45 | 6.78±1.23 |  |  |  |  |  |
| FEF25/75  (pred %) | N0 | 3.97±1.00 | .195 | .707 | .147 |  |  |
|  | N25 | 4.05±1.02 |  |  |  |  |  |
|  | N45 | 4.02±1.12 |  |  |  |  |  |
| FET (sec) | N0 | 1.90±0.51 | .018 | .982 | .002 |  |  |
|  | N25 | 1.88±0.66 |  |  |  |  |  |
|  | N45 | 1.91±0.56 |  |  |  |  |  |
| FVC: Forced Vital Capacity, PEF: Maximum Expiratory Flow Rate, FEV1: Forced Expiratory Volume İn 1 Second, PEF: Peak Expiratory Flow, FET: Forced Expiratory Time, N0: No-nap control, N25: a 25-minute nap, N45: a 45-minute nap, * p<.05 | | | | | | | |
